# Adaptability to changes in temporal structure is fornix-dependent

**DOI:** 10.1101/lm.038851.115

**Published:** 2015-08

**Authors:** Sze Chai Kwok, Anna S. Mitchell, Mark J. Buckley

**Affiliations:** 1 Key Laboratory of Brain Functional Genomics, Ministry of Education, Shanghai Key Laboratory of Brain Functional Genomics, Institute of Cognitive Neuroscience, School of Psychology and Cognitive Science, East China Normal University, Shanghai 200062, China; 2 NYU-ECNU Institute of Brain and Cognitive Science, NYU-Shanghai University, Shanghai 200062, China; 3 Neuroimaging Laboratory, Fondazione Santa Lucia, Istituto di Ricovero e Cura a Carattere Scientifico (IRCCS), Rome 00179, Italy; 4 Department of Experimental Psychology, University of Oxford, Oxford OX1 3UD, United Kingdom

## Abstract

Recognition memory deficits, even after short delays, are sometimes observed following hippocampal damage. One hypothesis links the hippocampus with processes in updating contextual memory representation. Here, we used fornix transection, which partially disconnects the hippocampal system, and compares the performance of fornix-transected monkeys with normal monkeys on two versions of a delayed-matching-to-position task with short delays. Spatial recognition memory was affected by fornix transection only when the temporal structure of the task changed across trials, while differences in motor control, motivation, perception, or short-term memory were not critical. We attributed the deficit to a compromised ability in tracking changes in task temporal structure.

Fornix transection disconnects one of the major input/output pathways of the hippocampus (Saunders and Aggleton 2007). In humans, fornix damage may result in long-term memory loss in recognition (Gaffan and Gaffan 1991; Poreh et al. 2006) and recall (McMackin et al. 1995; Aggleton et al. 2000) whereas short-term/working memory (STM) is sometimes spared (Scoville and Milner 1957; Cave and Squire 1992; Ryan and Cohen 2004). Similarly, monkeys with lesions of the hippocampal formation performed normally on a delayed nonmatching-to-sample task when the delays were short (≤15 sec) but were impaired at longer delays (>30 sec) (Alvarez et al. 1994), in keeping with the notion that the hippocampal system contributes to the recovery of contextual details in long-term memory (Tsivilis et al. 2008; Vann et al. 2009). However, other evidence has challenged this dissociation (Ranganath and Blumenfeld 2005; Kwok and Macaluso 2015). For example, lesions in the medial temporal lobe (MTL) can impair retention of information across short delays (Jeneson et al. 2012) and the hippocampus exhibits persistent activity during STM maintenance (Chein et al. 2011), indicative of a hippocampal role in STM too. Indeed, some recent neuropsychological studies associate hippocampal disruption with deficits in spatial tasks placing high demands on spatial configural processes even in the absence of any delay (Lee et al. 2005, 2006), further refuting delay-dependent theories.

Recent studies have provided one possible account to reconcile these seemingly contradictory results. On the one hand, empirical data from animal studies link temporal order processing to an extended diencephalic–hippocampal system in both rodents and primates (Fortin et al. 2002; Charles et al. 2004), suggesting the hypothesis that hippocampal networks mediate associations between sequential events. On the other hand, human neuroimaging studies have indicated that multivoxel patterns in the hippocampus contain temporal information (Hsieh et al. 2011; Ezzyat and Davachi 2014) and that interevent associative encoding and retrieval is mediated by the hippocampus (DuBrow and Davachi 2014), suggesting that hippocampal processes may play an important role in establishing temporal relationships among events. Pertinently, a recent fMRI study has further demonstrated that the hippocampus is very sensitive to the temporal duration characteristics of an event sequence and the interevent intervals, even on the order of seconds (Barnett et al. 2014).

These findings converged to suggest that the hippocampus is recruited for processing temporal duration information within sequences of events and the events’ interleaving intervals, and prompted us to hypothesize that memory impairment could be observed following hippocampal disruption (via fornix transection) when the temporal structure of events is subject to unpredictable alternation, even when delay intervals are short (a few seconds, cf. Barnett et al. 2014). We further predicted that such an impairment is not dependent on the delay intervals but rather on the animals’ ability in detecting and updating the temporal structure changes. Confirmation of this hypothesis can account for the absence of memory impairment in hippocampal lesioned monkeys at short delays when temporal structure was not manipulated (cf. Alvarez et al. 1994) and provide causal evidence for neuroimaging studies that have implicated the hippocampal system in integrating temporal structure information underlying episodic events.

To test the aforementioned hypothesis we used a two-alternative delayed matching to position paradigm wherein we manipulated the temporal structure of events by intermixing five different delay intervals so that the animals had to adapt across trials to the changing temporal structure across encoding and recognition. Critically, we restricted our investigation to very short delays (≤16 sec; cf. longer delays up to 120 sec were used in previous studies; Málková et al. 1995; Murray and Mishkin 1998). Behavioral data were acquired from 10 male macaque monkeys (*M* age 6.3 yr; *M* weight 8.3 kg). One group (FNX 1–3: all *Macaca fascicularis*) had received bilateral fornix transection and the remaining seven (CON 1–7: two *M. fascicularis* and five *M. mulatta*) acted as unoperated controls. Whereas hippocampal neurotoxic lesions often leave a relatively large proportion of hippocampal neurons intact (>25% on average; see Table 1 in Murray and Mishkin 1998), fornix transection can be assured of sectioning 100% of fornical fibers in every animal so this intervention is advantageous in its consistency despite its indirect effect upon hippocampal function. Certainly, fornix transection can be as effective as hippocampal lesions in producing deficits in some tasks such as delayed nonmatching-to-position tasks (Aggleton et al. 1992; Whishaw and Jarrard 1995). All licensed procedures were carried out in compliance with the United Kingdom Animals (Scientific Procedures) Act of 1986. A Home Office (UK) Project License obtained after review by the University of Oxford Animal Care and Ethical Review committee licensed all procedures. The animals were socially housed together in same species groups of between two and six animals. The housing and husbandry were in compliance with the guidelines of the European Directive (2010/63/EU) for the care and use of laboratory animals. Detailed description of the surgical procedure has been reported in Buckley et al. (2008). Microscopic examination of the stained sections revealed in every case a complete section of the fornix (Fig. 1B–D). A section of a control monkey's intact fornix is shown for comparison (Fig. 1A). The testing was performed in an automated apparatus, which consisted of a touch-sensitive screen (380 × 280 mm) on which visual stimuli were displayed and which could be touched by the monkey from its transport cage to obtain food pellets (190 mg; P.J. Noyes, Lancaster, NH) consequent upon correct responses. The monkeys performed one session per day, 6–7 d per week.

**Figure 1.**
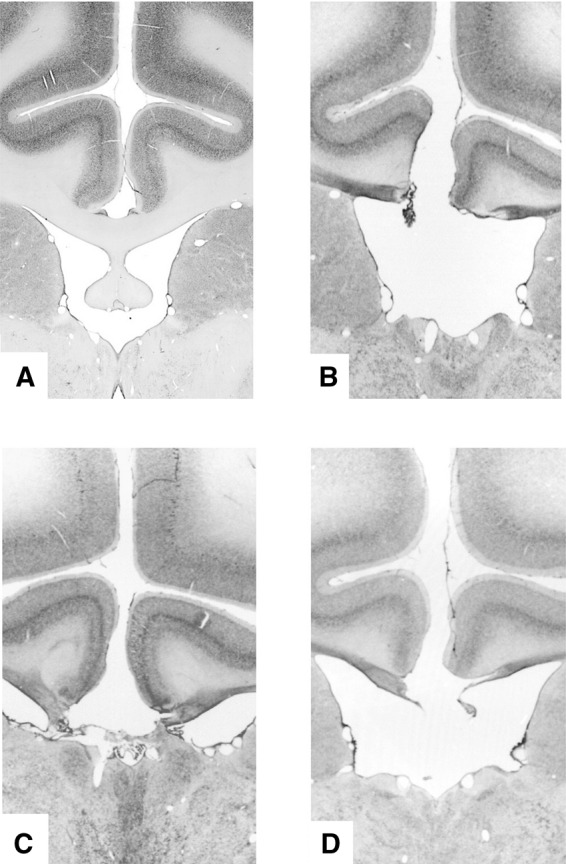
Histology of lesion. Coronal section from the brain of a normal unoperated macaque just posterior to the level of the interventricular foramen (*A*), and from the brains of the three operated monkeys showing that the fornix transection was complete (*B*–*D*).

All monkeys completed preliminary training (“task acquisition”) before performing the experiments described here (“Exp. 1,” “Exp. 2a–b”; one rhesus monkey in the CON group did not participate in Exp. 2b). We conducted Exp. 2 immediately after Exp. 1; no extra training was specifically administered prior to Exp. 2. For the lesioned animals, the task was administered post-operatively (on average ∼22 mo post-lesion). Each trial consisted of an encoding phase in which a spatial position (“sample”) was indicated by a red cross. After the monkey touched the sample, a blue square (“distractor”) appeared in the center. A touch to the blue square initiated a variable delay interval (i.e., the manipulation for Exp. 1) and then a choice phase consisting of two identical red crosses in different positions. One of the crosses appeared in the same position as the sample (target; S+) and the other one in a different position (foil; S−). A touch to the S+ resulted in a delivery of a reward pellet, removed the S−, and the S+ remained alone for a further 1 sec for positive feedback. The screen would then be blanked for an ITI of 6 sec before the next trial (or an ITI of 12 sec following a touch to S−). There was no time constraint imposed on responses made to the probe choices and therefore there were no missed trials. We also did not repeat the same problem to the monkey following a mistake; each trial was new, and independent of the outcome of the preceding trial. The sample subtended a visual angle of ∼9° in task acquisition and Exp. 1, or ∼6.8° in Exp. 2a–b. The distractor subtended a visual angle of ∼4.6° in all experiments.

During task acquisition the monkeys were trained until they reached a ≥90% performance level within a 100-reward session. All trials in this stage consisted of a short delay interval (1 sec), and a wide separation between choice positions (21.7°; or 23 cm) to make the trials “easy.” Upon reaching criterion, the two groups were not different in the number of errors accrued, *t*_(8)_ < 1, number of rewards received, *t*_(8)_ < 1, and number of sessions performed, *t*_(8)_ < 1, indicating that the FNX monkeys learned to perform these spatial recognition problems as well as controls. Indeed, specifically in the last 100-reward training session, there was no difference between the two groups, *t*_(8)_ < 1. This suggests that when conditions were equated on both spatial and temporal-delay difficulty—and critically, without intermixed delays—FNX performed as well as CON monkeys. Exp. 1 consisted of two consecutive daily sessions. Each animal worked for 200 rewards in total (but accrued varying numbers of errors). Trials within a session were divided into five trial-types with differing intervals of delay [either 1, 2, 4, 8, or 16 sec] between the distractor and probes. The trial-type order was randomized within each successive set of five trials (with one trial of each trial-type per set) so that the delay changed unpredictably from one trial to another. The two probe choices were separated by a visual angle of 21.7°.

We planned to analyze raw errors per trial-type, but such analysis of errors per trial-type necessitates equating the total number of correct responses accrued per trial-type and in our design each animal worked for 100 rewards (i.e., correct responses) in each session albeit without any constraint on the maximum correct responses/rewards that could be accrued for each of the five trial-types. Given this limitation we first sought to analyze the maximum possible amount of data that allowed equal rewards per trial-type to be considered. To do this we calculated the maximum number of correct responses that all animals had made to all trial-types—which were 27 and 24, respectively for Exp. 1 and Exp. 2 (see Table 1)—and then calculated how many errors were accrued in each trial-type for these equated numbers of rewards per trial-type. This analysis only involved discarding a small proportion of data (we included 70% of all trials for Exp. 1, and 67% and 66% for Exp. 2a and Exp. 2b, respectively [averaged across animals, see Table 1]). We recorded response time (RT) for every trial and ascertained that RT for the distractor touch was short (*M* 935 ms, *SD* 292 ms), and did not differ between groups, *F*_(1,8)_ = 2.36, *P* = 0.163, or between delay conditions, *F*_(4,32)_ = 1.50, *P* = 0.226, confirming that any effect on performance was not due to the animals in either group being differentially distracted or differentially willing/unwilling to initiate the required touch to the distractor.

**Table 1.**
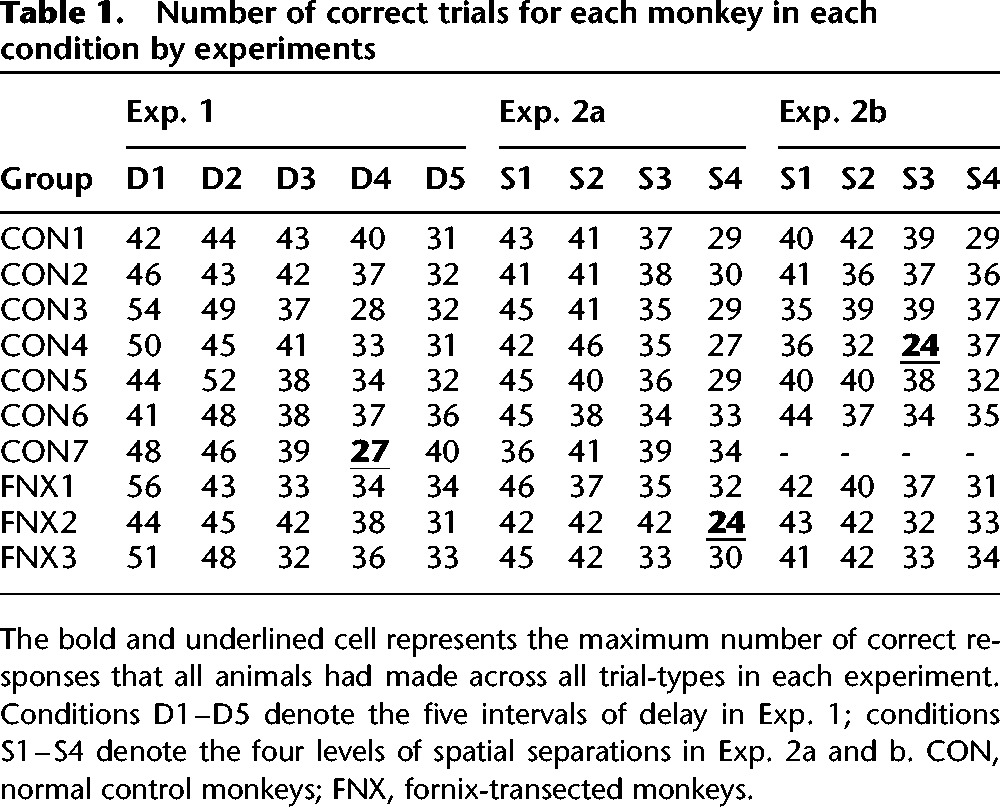
Number of correct trials for each monkey in each condition by experiments

The results on errors for Exp. 1 are presented in Figure 2A, which shows that the number of errors increased with the delay duration for both groups and the FNX group made more errors throughout. An ANOVA with two levels of the between-subjects factor (“Group”: CON, FNX) and five levels of the within-subjects factor (“Delay”: 1, 2, 4, 8, and 16 sec) on the number of errors revealed main effects of “Group,” *F*_(1,8)_ = 6.72, *P* = 0.032, and “Delay,” *F*_(4,32)_ = 17.19, *P* < 0.001, but no “Group × Delay” interaction, *F*_(4,32)_ = 1.06, *P* = 0.394. The results were not due to the mixed species in the CON group because an analysis that only considered cynomolgus monkeys, by excluding the five rhesus monkeys in the CON group, produced the same outcome, “Group,” *F*_(1,3)_ = 128.16, *P* = 0.001. In light of the aforementioned analysis not considering a small subset of the data we also verified if the main results would be the same when all the trials were included. The patterns of results were the same as our main analyses when considering all trials, for Exp. 1, we confirmed the main effects of “Group,” *F*_(1,8)_ = 5.30, *P* = 0.050, and “Delay,” *F*_(4,32)_ = 22.15, *P* < 0.001, and no “Group × Delay” interaction, *F*_(4,32)_ = 1.04, *P* = 0.404. For completeness, we also performed a percentage correct analysis which likewise considered all the data albeit with the drawback that our experimental design leads to sampling bias as data-gathering for all trial-types stops whenever the easiest trial-type or trial-types accrue enough corrects. Nonetheless, in a one-tailed *t*-test, this analysis also revealed significance for the main effect of “Group” in Exp. 1, *F*_(1,8)_ = 4.05, *P* = 0.039, again supporting our hypothesis.

**Figure 2.**
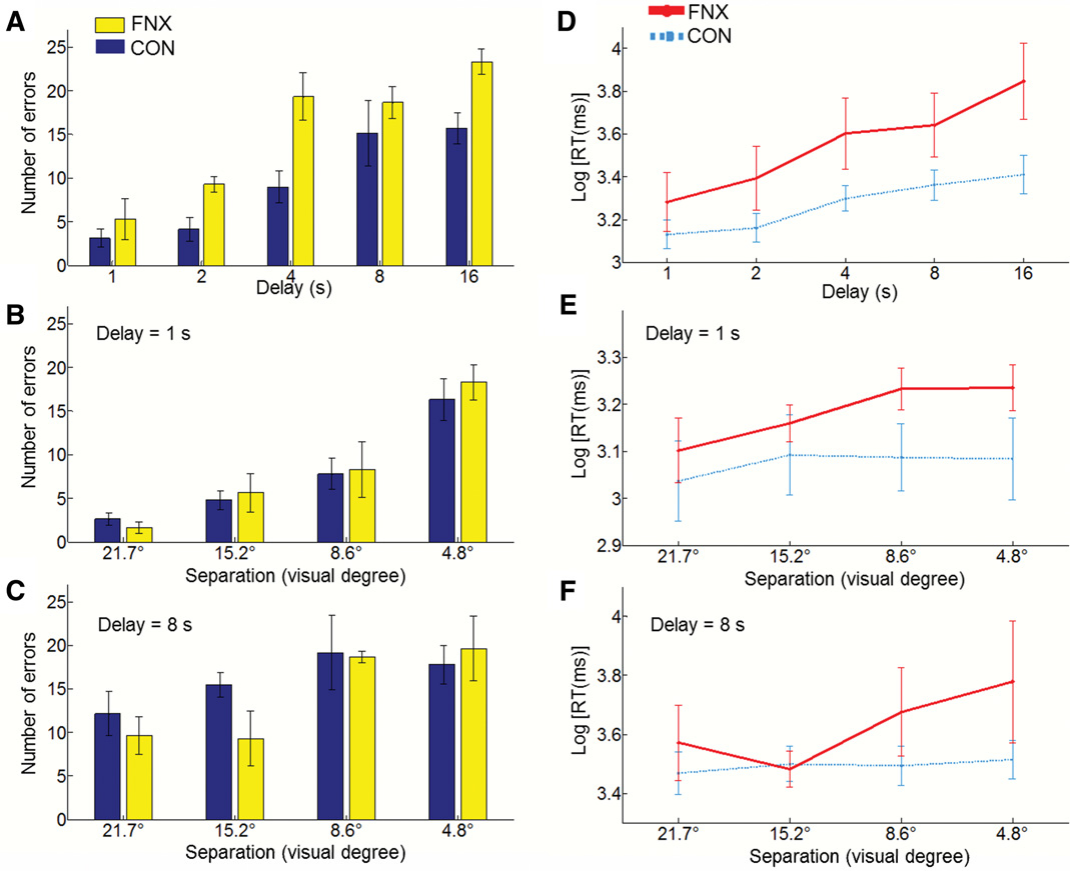
Recognition performance and response time for fornix transected (FNX) and control (CON) groups. (*A*–*C*) Recognition performance expressed as number of errors by levels of conditions in Exp. 1 (*A*), Exp. 2a (*B*), and Exp. 2b (*C*). (*D*–*F*) Response time (RT) expressed as logarithmically transformed RT by levels of conditions in Exp. 1 (*E*), Exp. 2a (*D*), and Exp. 2b (*F*). The log-RT plots include both correct and error trials. Error bars depict the standard error of the means.

Additionally, we performed the following analyses to further test for a possible delay-dependent impairment in the FNX group. First, we reduced the number of levels from five to two by collapsing the delays at either extreme. We then re-ran the analysis on these two levels, with a crossing by “Group,” to compare the mean number of errors in the cells of 1-, 2-, and 4-sec delays (“short” delays) versus the mean number of errors in the cells of 8- and 16-sec delays (“long” delays) in the two groups. Again, we replicated the main effects of “Group,” *F*_(1,8)_ = 5.55, *P* = 0.046, and of “Delay,” *F*_(1,8)_ = 55.49, *P* < 0.001, but no “Group × Delay” interaction, *F* < 1. As a control, we ran pairwise comparisons comparing the two groups at the “short” and “long” levels, and found the effect to be stronger in the “short,” *t*_(8)_ = 3.85, *P* = 0.005, than in the “long” delay condition, *t*_(8)_ = 1.55, *P* = 0.161. As a further constraint, we obtained the same results even by excluding all the intermediate-delay trials (4-sec trials) from the analysis (i.e., comparing only 1-sec/2-sec versus 8-sec/16-sec). We again found no “Group × Delay” interaction, *F* < 1, with pairwise comparisons showing that the effect was significant in the “short” delay condition, *t*_(8)_ = 2.33, *P* = 0.048, but not in the “long” delay condition, *t*_(8)_ = 1.55, *P* = 0.161.

In order to check whether the FNX lesion-related effect might have stemmed from the longer-delay conditions, we performed pairwise comparisons comparing the two groups at each level of the “Delay” factor for Exp. 1. The FNX group was significantly worse in three (out of five) delays: delay of 2 sec, *t*_(8)_ = 2.36, *P* = 0.046; delay of 4 sec, *t*_(8)_ = 3.13, *P* = 0.014; and delay of 16 sec, *t*_(8)_ = 2.62, *P* = 0.031, but not for delay of 1 sec, *t*_(8)_ = 1.02, *P* = 0.338; and delay of 8 sec, *t*_(8)_ < 1. The pairwise comparisons as a whole did not show a consistent pattern/trend that the impairment was tilted toward the longer delays despite the comparison at the shortest delay (1 sec) not revealing a statistically significant difference between FNX and CON groups. Instead, FNX monkeys were impaired in the two relatively short delays at both 2 and 4 sec (but not at the longer 8-sec delay), refuting the possibility that FNX monkeys were simply worse at longer delays. This set of additional tests lends further support to the argument that FNX monkeys were impaired even at the relatively short-delay conditions and the impairment did not exacerbate as the delays increased. This confirmed that the FNX group was impaired relative to CON in remembering the sample locations across all delay intervals and that the effect of fornix transection on memory recognition was not delay-dependent. However, we caution that our argument of “delay-independency” should be interpreted more narrowly within the range of relatively short delays (note that the longest delay here was just 16 sec). This qualifier might partially account for the inconsistency with a previous study where the authors found an interaction between delay and hippocampal lesion in accuracy in a delayed nonmatching to sample task when the delays were changed much more drastically between 1, 60, and 600 sec (Alvarez et al. 1994).

We analyzed the response time using log-transformed RT data. For Exp. 1, an ANOVA on the log-transformed RT data for responding to the probe revealed a main effect of “Group” with a trend toward significance, *F*_(1,8)_ = 4.75, *P* = 0.061, but without a “Group × Delay” interaction, *F*_(4,32)_ = 1.80, *P* = 0.154 (see Fig. 2D). The equivalent RT analysis using correct trials revealed a main effect of “Group,” *F*_(1,8)_ = 5.44, *P* = 0.048, and only a marginally significant “Group × Delay” interaction, *F*_(4,32)_ = 5.40, *P* = 0.049. Although the FNX monkeys responded slightly slower across all trial-types, in the present analyses, we were not able to completely disentangle the respective processes underlying RT and accuracy (cf. RT results in Exp. 2).

This pattern of impairment supports our hypothesis that memory recognition that required adapting to a changing temporal structure with “variable delays” across trials is affected by disruption to the hippocampal system. Indeed, the altered task structure (when animals transitioned from the easy acquisition phase to the more demanding Exp. 1 and were exposed to five different delay lengths intermixed throughout the session) was not trivial because it required animals to learn about the changing temporal structure of trials, for example, in distinguishing intra- from intertrial intervals, and in adjusting the maintenance duration from one trial to the next (e.g., from 1 sec → 16 sec). Previous studies have shown that fornix transection impairs learning and memory in the temporal domain (Charles et al. 2004; Brasted et al. 2005) and the deficit here may similarly reflect an impairment in learning about temporal structure (Wilson et al. 2007). Here, our findings confirmed such a prediction and provided causal evidence for previous studies which suggested that the hippocampus is involved in integrating interval duration information contained within a sequence (Barnett et al. 2014). However, one might also argue that the impairment was caused by the spatial demands (e.g., Murray et al. 1989; Buckley et al. 2004), and/or “general” contextual change (i.e., not temporal specific). Therefore we conducted two control experiments (Exp. 2a–b) to rule out these alternative explanations.

In Exp. 2, we increased the spatial demand and introduced a (nontemporal) change of context across trials. Specifically, we modulated the difficulty of spatial discrimination by modulating the spatial separation between choice items in a nonpredictable manner between trials. Trials within a session were divided into four different trial-types with differing spatial separations (visual angles of either 4.8°, 8.6°, 15.2°, or 21.7° [equivalent to 5, 9, 16, and 23 cm]) between probe choices. Sets of four trials containing one of each of the trial-types were presented, with the trial-type order randomized within each set. This effected changes in spatial context (cf. fixed separation of 21.7° in Exp. 1) while keeping the delay interval constant at either 1 or 8 sec in Exp. 2a and 2b, respectively. Each control experiment comprised one single session, each requiring the animal to accrue 150 rewards.

In order to compare performance across trial-types in Exp. 2 we calculated the maximum number of correct responses that all animals had made to all trial-types, which was 24. We then analyzed the raw numbers of errors that animals made in accruing these first 24 correct responses per trial-type. Again, the time taken to touch the distractor was short (Exp. 2a: *M* 982 msec, SD 407 msec; Exp. 2b: *M* 1361 msec, SD 581 msec) and did not differ between groups (both *F* < 1), or between conditions (both *F* < 1). An ANOVA with two levels of “Group” (CON, FNX), four levels of “Separation” (4.8°, 8.6°, 15.2°, and 21.7°) and two levels of “Delay” (1, 8 sec) on the number of errors revealed no effect of “Group,” *F*_(1,7)_ < 1, and no interaction involving “Group”: “Group × Delay,” *F*_(1,7)_ = 1.13, *P* = 0.323; “Group × Separation,” *F* < 1; and “Group × Delay × Separation,” *F* < 1. The main effects of “Delay,” *F*_(1,7)_ = 38.41, *P* = 0.001, “Separation,” *F*_(1,7)_ = 19.59, *P* < 0.001, and their interaction, *F*_(1,7)_ = 4.08, *P* = 0.019 were expected and beyond the motivation of Exp. 2. In order to check if the main results would be replicated when all the trials are included, we analyzed data from all trials for Exp. 2. The analysis confirmed the main findings that there were no main effects of “Group,” *F*_(1,7)_ < 1, and no interactions involving “Group”: “Group × Delay,” *F*_(1,7)_ < 1; “Group × Separation,” *F*_(3,21)_ = 21.16, *P* = 0.067; and “Group × Delay × Separation,” *F* < 1. For completeness, we also performed a percentage correct analysis but no significant main effects or interactions involving “Group” were found, all *F* < 1. To further illustrate the lack of a lesion-related impairment in Exp. 2, we performed pairwise comparisons comparing the two groups at each level of “Separation”; none but one pairwise comparison was significant: *t*_(7)_ < 1. In that exception (spatial separation of 8.6°), the CON group was actually worse than the FNX group, *t*_(7)_ < −3.37, *P* = 0.012 (Fig. 2B–C).

An ANOVA on the log-transformed RT data for responding to the probe did not disclose a main effect of “Group,” *F*_(1,7)_ = 1.45, *P* = 0.263, nor for two of its interaction terms: “Group × Delay,” *F* < 1; “Group × Delay × Separation,” *F* < 1. The “Group × Separation” interaction was however significant, *F*_(3,21)_ = 4.20, *P* = 0.018 (see Fig. 2E–F). This interaction term hints that the FNX lesion effects on accuracy and RT might be dissociable. The equivalent RT analysis using correct trials revealed the same pattern of results.

In Exp. 2, FNX monkeys were not impaired across any or all levels of spatial separation, and together with the lack of difference in task acquisition, we could rule out explanations for the deficit in Exp. 1 in terms of impaired motor control, motivation, spatial perception, short-term retention, or “fast-learning” deficits (Kwok and Buckley 2010; cf. Kwok and Buckley 2009). These results demonstrated that a nontemporal contextual change was insufficient to cause mnemonic deficits as observed in Exp. 1.

Finally, in order to ascertain that the manipulation of intermixing variable delays was indeed the main cause for the impairment in the FNX monkeys, we performed a “cross-experiment” analysis to directly compare the two experiments. We averaged the number of errors across all levels of conditions in each of the experiments and entered these values into a repeated-measures ANOVA with two levels of the between-subjects factor (“Group”: CON, FNX) and only two levels of a within-subjects factor (“Exp”: intermixed, blocked). We found a strong “Group × Exp” interaction, *F*_(1,7)_ = 51.45, *P* < 0.001, with the impairment being stronger in Exp. 1 (note that the one CON animal which did not participate in Exp. 2b was excluded from this analysis); no main effects of “Group” or “Exp.” were found, with both *P* > 0.05. We also repeated this analysis using data extracted only from the 1- and 8-sec conditions in Exp. 1 so as to equate the conditions in the “blocked-delay” experiments, and confirmed the same “Group × Exp.” interaction, *F*_(1,7)_ = 10.29, *P* = 0.015, again with the impairment being stronger in the fornix group. This interaction effect can also be seen by contrasting Fig. 2A with Fig. 2B–C. Notably, this analysis showed that the deficit in the FNX monkeys was significantly more severe in Exp. 1 (where delays were intermixed) than in Exp. 2 (where delays were blocked), indicating that the impairment was indeed related to the variable delays in the intermixed condition.

Focusing on the effects of changing temporal structure across trials, the current findings on the role of the hippocampus contrast with those that have examined the hippocampal mechanisms for integrating disparate elements of an experience during memory formation. For example, the “temporal discontiguity” in Staresina and Davachi (2009) refers to how the object information and its associated featural information (colour) were separated in time during encoding, whereas our current study manipulated the intra- and intertrial temporal delays between encoding and retrieval. The underlying cause of the impairment in our study is not attributed to temporal discontiguity between integrating different constitutive elements of a single event at encoding, rather, we offer a mechanistic account in terms of a lesion-induced deficit resulting in diminished “flexibility” in monitoring and/or adapting to the temporal changes/unpredictability across many different events. The selective effect on our Exp. 1 is reminiscent of hippocampal involvement in associatively linking temporally separated events (Rawlins et al. 1985). This is especially related to recent findings showing that the human hippocampus is responsive to the detection of changes in temporal duration within a sequence of events, for example, on detecting how much time elapsed between pairs of events within the sequence (Barnett et al. 2014). By inference, if the hippocampal system is compromised, such sensitivity to detect—and adapt to—the changes in temporal duration separating the events should be affected.

Our findings share important links with an interpretation in which hippocampal representations reflect the statistics of the environment, consistent with its role in learning statistical task structure (Bornstein and Daw 2012) and in incidental learning of temporal regularities (Schapiro et al. 2012, 2014). This perspective accords with a fundamental hippocampal function in relational/configural learning (Cohen and Eichenbaum 1993), even after a very short delay (Olson et al. 2006), which comprises not only relating events occurring simultaneously in an episode, but also discovering event relations obtained stochastically between temporally discontiguous events (Shohamy et al. 2009; Hales and Brewer 2010). Learning a probabilistic transition structure, such as imputing the equivalence relationships in Shohamy et al. (2009), requires integrating events across time rather than within an episode (Ezzyat and Davachi 2014).

In summary, we conclude that general impairments in spatial recognition memory after short delays are not a necessary consequence of hippocampal system disruption; rather, one also needs to consider whether a compromised ability to track changes in temporal structure might be more relevant. Given that the fornix also carries some projections that do not terminate in the hippocampus (Saunders et al. 2005), we acknowledge that the observed effects might have contributions from disruption in signals supported by other nonhippocampal MTL cortices such as the entorhinal cortex (Sugase-Miyamoto and Richmond 2007), the medial prefrontal cortex (Cross et al. 2013), and/or the medial thalamus (Mitchell and Dalrymple-Alford 2005).
